# Diffuse Alveolar Damage: A Common Phenomenon in Progressive Interstitial Lung Disorders

**DOI:** 10.1155/2011/531302

**Published:** 2010-11-02

**Authors:** Riitta Kaarteenaho, Vuokko L. Kinnula

**Affiliations:** ^1^Respiratory Research Unit, Department of Internal Medicine, Centre of Excellence in Research, and Clinical Research Center, Institute of Clinical Medicine, Oulu University Hospital, University of Oulu, P.O. Box 22, 90029 Oulu, Finland; ^2^Pulmonary Division, Department of Medicine, Helsinki University Hospital, University of Helsinki, P.O. Box 22, 00014 Helsinki, Finland

## Abstract

It has become obvious that several interstitial lung diseases, and even viral lung infections, can progress rapidly, and exhibit similar features in their lung morphology. The final histopathological feature, common in these lung disorders, is diffuse alveolar damage (DAD). The histopathology of DAD is considered to represent end stage phenomenon in acutely behaving interstitial pneumonias, such as acute interstitial pneumonia (AIP) and acute exacerbations of idiopathic pulmonary fibrosis (IPF). Acute worsening and DAD may occur also in patients with nonspecific interstitial pneumonias (NSIPs), and even in severe viral lung infections where there is DAD histopathology in the lung. A better understanding of the mechanisms underlying the DAD reaction is needed to clarify the treatment for these serious lung diseases. There is an urgent need for international efforts for studying DAD-associated lung diseases, since the prognosis of these patients has been and is still dismal.

## 1. Introduction

Acutely behaving interstitial lung diseases (ILDs) comprise several diseases with both known and unknown causes. A common feature in these acute manifestations is the appearance of diffuse alveolar damage (DAD) in the lung, which is generally associated with very bad prognosis and high mortality of the patients. An acute ILD may be a rapidly evolving idiopathic or nonidiopathic disease, it may occur as the first symptom or it may represent a predilection of a chronic lung disease, or alternatively, it can be encountered as an acute manifestation of a chronic ILD. The pathogenetic mechanisms underlying each of these disorders and their acute exacerbations are largely unknown. From a clinical point of view, the diagnostics and treatment of these patients are very demanding, since in addition to the differential diagnostics mentioned above, diagnostic procedures have to exclude infections, drug reactions, and other diseases such as cardiac diseases and pulmonary embolisms and other clinical manifestations resembling acute respiratory distress syndrome (ARDS), such as diffuse alveolar hemorrhage (DAH) and acute fibrinous organizing pneumonia (AFOP). Moreover, the nomenclature of these diseases has changed in the past decade and thus it is not uncommon that many specialists, for example pulmonologists, pathologists, radiologists and intensive care specialists, may refer to the same disorder by different terms. Patients suffering from aggressive ILDs are very challenging for many specialists, not least for pulmonologists also due to the appearance of unpredictable acute disease exacerbations.

## 2. DAD as a Pathological Phenomenon in the Lung

First studies on DAD have been published over 40 years ago, but the phenomenon is still a mystery. In one of the first studies on this issue Cederberg et al. described hyaline membranes at autopsy of patients who had received oxygen at high concentrations [1]. Although the histological features of DAD were described even earlier, the name of diffuse alveolar damage, that is, DAD, was presented by Katzenstein and co-authors in their comprehensive review article, which included also their own data of over 400 patients with DAD and role of oxygen, shock, and related factors [2]. They concluded that DAD is a nonspecific reaction of the lung to a multitude of injurious agents, and that endothelial and alveolar cell injury leads to fluid and cellular exudation, with hyaline membranes and edema being well-known features. A few years later Askin & Katzenstein reported that pneumocystis infection may masquerade DAD [3]. A later study revealed autopsy findings of 17 patients with acquired immunodeficiency syndrome (AIDS) of which most had DAD in their lungs cytomegalovirus and/or pneumocystis carinii being a most likely etiology [4]. One of the first immunohistochemical studies on this issue in humans was published in 1995 when a study by Matsubara and co-authors showed destruction of the alveolar basement membrane in DAD [5]. Studies on matrix metalloproteinases (MMPs) and their tissue inhibitors (TIMP) in DAD and idiopathic pulmonary fibrosis (IPF) demonstrated that both factors are associated with myofibroblasts and epithelial cells [6]. Subsequent study revealed the presence of *α*-smooth muscle actin positive myofibroblasts in the proliferative phase of DAD [7]. Hyaline membranes of patients with DAD showed such a heterogeneity, which may suggest that these structures might be formed by different mechanisms in DAD of various aetiologies [8]. To conclude, the studies of the histopathology and pathogenesis of DAD in human lung are sparse, and so far very little has been learnt about its pathogenesis after the review article of Katzenstein and co-authors which was published nearly four decades ago.

## 3. Idiopathic Interstitial Pneumonias That May Behave Acutely with DAD Histopathology

Idiopathic interstitial pneumonias (IIP) comprise a disease group that belongs to the ILDs. The classification of IIPs has changed several times within the past five decades [9, 10]. In the current classification, the histological pattern and clinico-radiographic-pathologic diagnosis are named with different terms [11] (Table 1). The reason for this kind of double nomenclature system is that many histologic patterns are presented not only in idiopathic diseases, but also are associated with other disorders such as connective tissue, that is, collagen vascular diseases (CVD) and drug reactions. Since the clinical behaviour of the idiopathic and nonidiopathic forms of IIPs may be different, it is reasonable to define these diseases clinically with other terms. In the present review, in particular IPF with the histopathology of usual interstitial pneumonia (UIP), nonspecific interstitial pneumonia (NSIP) and acute interstitial pneumonia (AIP) are described since all of them can behave aggressively and may be associated with DAD in the lung (Table 2). It should be stressed, however, that DAD is more frequent in the patients with IPF than in NSIP. The other types of interstitial pneumonias generally follow a more benign course and have not therefore been included in this paper. In addition there is a recent review on acute exacerbations of interstitial lung diseases, but that paper included also sarcoidosis and vasculitides and detailed suggestions for the treatment of these disorders [12]. Sarcoidosis and vasculitis are now excluded due to the systemic manifestations of those disorders. 

### 3.1. IPF

IPF is the most common IIP, accounting for about 25% of the all interstitial pneumonias [11]. In Finland, its prevalence has been estimated to be 16–18/100000, being more common in males aged 50–70 years than in females [13]. It is usually a progressive disease with the average survival being no more than 2–4 years after diagnosis [14]. No efficacious medical treatment is currently available, and the only potential cure may be lung transplantation (Table 2). Aggregates of fibroblasts and myofibroblasts, that is, fibroblast foci (FF) are a characteristic of UIP, even though these cell types may be present also in other types of pulmonary fibroses (e.g., CVDs and asbestosis) to a lesser extent [15]. Some of the patients experience acute exacerbation (AE) with concurrent DAD histopathology that significantly worsens the prognosis. Preventing, predicting and treating these AEs are key issues in the long-term followup of these patients, but unfortunately all are currently unresolved.

### 3.2. NSIP

NSIP was described in 1995 by Katzenstein and Fiorelli [16]. It is a disease that may be idiopathic, although it is the most common type of interstitial pneumonias associated with many CVDs [17]. Patients with NSIP are usually younger than their counterparts with IPF, and it occurs more often in women than in men. Children may also suffer NSIP as an idiopathic disease or as a CVD-associated disease [18]. NSIP may progress slowly or it can present as a nonprogressing disease; it may also respond to corticosteroid treatment and also in that respect it differs from IPF. Most, that is 74%–82%, of NSIP patients are still alive 5 years after diagnosis [19, 20]. NSIP has been divided into cellular and fibrosing patterns, of which the latter may resemble IPF in its clinical course and prognosis [21]. The histology of NSIP is usually uniform. In the cellular type of NSIP, chronic interstitial inflammatory cells involves the alveolar walls, whereas the fibrotic form of NSIP may include more advanced fibrosis [16, 21]. The prognosis of the fibrotic type of NSIP is dismal, and the problems encountered including AE and DAD are very similar as in the above described disease, IPF.

### 3.3. AIP

**Figure 1:**
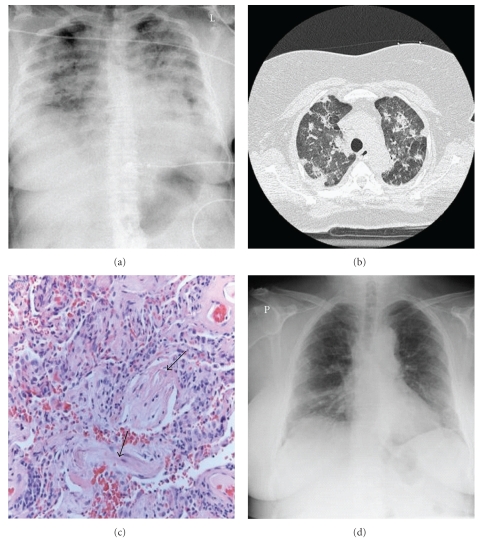
Radiological and histological findings of an AIP patient who was actively treated by mechanical ventilation and high doses of corticosteroids and immunosuppressive drugs. (a) Chest X-ray showing alveolar infiltrates at the time when the patient was mechanically ventilated. (b) HRCT findings of the upper lung lobes showing ground glass opacity and infiltrates. HRCT was taken on the following day after the chest X-ray shown in panel (a) Both Chest-X-ray and HRCT have been taken 2-3 days before taking the lung biopsy by VATS. (c) Lung biopsy findings of the same patient showing interstitial fibrosis and fibroblast proliferation representing late stages of organizing phase of DAD after treatment of several weeks. The patient recovered well with minimal radiological (d) and functional abnormalities within the lungs documented 4 years after the episode.

AIP was firstly described by Hamman and Rich in 1935 [22]. For many decades the patients in this first paper were assumed to have been suffering from UIP/IPF, in fact, it was 1990 when these patients were shown to truly suffer from AIP [23]. Clinically AIP presents as a rapid development of respiratory failure in a previously healthy person [24, 25]. Both clinically and radiologically, it usually displays the features of ARDS, but in contrast to ARDS, no specific cause as an aetiology can be found, therefore it could be considered as “idiopathic ARDS” [26, 27] (Figure 1(a)–1(d)). The histological pattern of AIP is typically DAD with hyaline membranes, edema, and interstitial acute inflammation [28]. In the organizing phase of DAD (approximately 5–10 days after injury), alveolar septal thickening with loose organizing fibrosis, type II pneumocyte hyperplasia, and patchy or diffuse airspace organization can be found (Figure 1(c)). The prognosis of AIP is dismal and the mortality high. It has been suggested that by adopting an aggressive diagnostic approach, that is, mechanical ventilation with a lung-protective strategy, and the early institution of immunosuppressive therapy it may be possible to improve the clinical outcome of AIP [29–31]. Controlled prospective studies on the treatment of AIP are not available, not surprising since AIP is so uncommon and it is generally a very acute lung manifestation displaying some overlapping features with ARDS.

## 4. Acute Exacerbation of IPF

Although IPF is usually a relentlessly, progressive disease lasting for months, even years, it may also start with an insidious onset and display AEs, in which the typical features are worsening dyspnea, cough, low-grade fever, worsening gas-exchange parameters, and the appearance of new opacities in radiology [32, 33]. This condition needs to be differentiated from AIP, in which the patients have not had a previous lung disease. There are no universal accepted diagnostic criteria for AE of IPF, although the criteria have been proposed by Kondoh, Akira, and Kim and most recently the IPF Clinical Research Network Investigators (Table 3) [32–35], that is, worsening of dyspnoea within one month and deterioration of gas-exchange parameters, combined with the exclusion of heart diseases, pulmonary embolisms, and infections (Table 3). A recent study showed that one-year and three-year incidence of AE of IPF was 14.2% and 20.7%, respectively [36].

The etiology of AE of IPF is unknown, although some published case reports have proposed that bronchoalveolar lavage (BAL) or lung biopsy procedures, or certain drugs or treatments might trigger AE in some IPF patients [37, 38]. A recent study claimed that in patients with IPF, the presence of ongoing AE at the time of the lung biopsy surgery represented a marked risk factor for complications and mortality [39]. Circulating fibrocytes have been shown to be elevated in patients with stable IPF, with a further increase occurring during AE of IPF, whereas the patients with ARDS were not different from healthy control subjects or stable patients with IPF [40]. Another recent study observed that a total of 579 genes were differentially expressed between stable IPF and AE of IPF, however a functional analysis of these genes did not reveal any evidence of an infectious or inflammatory aetiology [41]. Overall, the results obtained from the gene expression profiles identified epithelial injury and proliferation as the key molecular genetic events in AE and proposed that plasma defensins could be considered as new biomarkers for the acute disease exacerbation. A quite recent study by Collard and co-authors interestingly showed that plasma from patients with AE of IPF showed significant elevations in markers of type II alveolar epithelial cell injury and/or proliferation, endothelial cell injury, and coagulation, and moreover, that this profile differed from the biomarker profile of the patients with acute lung injury [42]. 

AE of IPF presents with radiological changes in addition to the typical IPF-alterations, which especially in lower lungs, include peripheral predominant, basal predominant reticular abnormalities, with honeycombing and traction bronchiectasis and bronchiolectasis [43]. Akira and co-authors studied 58 IPF-patients with AE [44]. Two radiologists evaluated the features with high-resolution computed tomography (HRCT). In this evaluation, the overall extents of the parenchymal changes, consolidations, reticulation, and honeycombing were noted. The HRCT changes were classified into three types, namely, peripheral, multifocal, and diffuse patterns. That particular study showed that during the AE process the radiological and HRCT changes may vary, and that a peripheral pattern is a common phenomenon. The conclusion from the radiological studies is that it appears that the shortest survival of AE of IPF is associated with diffuse abnormality in HRCT [44].

Based on a systematic review of 8 published studies, each of which had rather small numbers of IPF patients with AE, most of the cases were men between 65–70 years, whose lung function tests were relatively good, suggesting that the clinical course of the disease had been at an early phase [33, 35, 38, 45–50]. Nearly all patients displayed neutrophilia in the BAL fluid, ground glass opacities, and consolidation in HRCT. Importantly all biopsied cases presented DAD superimposed on UIP lung histopathology. The prognosis of AE was poor in both the mechanically invasively and the noninvasively treated groups, since most of the patients had died within one month after the AE despite active medical treatment [45]. At present there is no consensus about the treatment of AE of IPF, although most patients receive therapy with corticosteroids and other immunosuppressive drugs. One study evaluating the use of pirfenidone in patients with IPF had to be terminated prematurely because in the placebo-control group the patients suffered more AEs than occurred in the pirfenidone group, indicating that the drug might have some efficacy in the prevention of AE of IPF [51]. These suggestions need to be confirmed in the future before any therapeutic conclusions can be drawn. Efforts in recognizing and predicting the exacerbation as early as possible are of major significance for the overall treatment of the patient, including the possibility of lung transplantation.

## 5. Acute Exacerbations in Other Interstitial Lung Diseases

In addition to IPF, AEs have been shown to occur in other types of ILDs. Based on one study of 74 patients with idiopathic NSIP and 93 patients who had both CVD and ILD, AE occurred in six patients with idiopathic NSIP and in four patients with CVD-ILD, that is, in rheumatoid arthritis and scleroderma [39]. In that study, AE was diagnosed by the Akiras criteria, and in two cases it took place immediately after surgical lung biopsy. Again the lung biopsy findings showed DAD in addition to NSIP. Another recent study examined patients with surgical lung biopsy proven fibrotic hypersensitivity pneumonitis that is, allergic alveolitis, who had experienced an acute decline in their respiratory status and who met criteria similar to those proposed for the diagnosis of an AE of IPF [52]. These patients experienced a clinical course similar to that of AE of IPF. All cases resulted either in death of the patient or lung transplantation. Lung biopsy at the time of AE or autopsy revealed organizing DAD superimposed on fibrotic lung disease [52–54]. In a recent study of 83 biopsied patients with CVD and ILD, five patients suffered from rheumatoid arthritis and one patient from scleroderma, which associated either with UIP, NSIP, or unclassifiable ILD change and AE. Five of these patients died despite active treatments that included invasive mechanical ventilation and immunosuppressive medication [55]. The prognosis of the patients with CVD is generally significantly better compared to IPF. It can, however, be concluded that AEs in these other interstitial lung diseases can exhibit very similar clinical, radiological, and histological findings. It needs to be emphasized also that the lung manifestations of systemic diseases are complex and therefore need to be evaluated very carefully.

## 6. Intensive Care Treatment and AE of ILDs

The development of DAD is still poorly understood, and most treatments are empiric. Several studies have indicated that IPF patients with acute lung failure do not benefit from intensive care treatment and invasive mechanical ventilation [56–61]. It has been also proposed that invasive mechanical ventilation should be used only in those patients who are scheduled to undergo a lung transplantation operation within a few days. When an ILD patient experiences an acute lung failure, there are many possible causes to be considered in the diagnostics for example, deterioration of chronic lung diseases, infections, AE of the disease, or drug reactions. Recent guidelines published by the British Thoracic Society recommend palliative treatment for the patients with AE of IPF, whereas those ILDs that are reversible, need to be diagnosed and treated actively [62]. Thus the reversible ILD with acute respiratory failure should be treated by intensive care treatment with mechanical ventilation, and thus BAL, transbronchial biopsy and surgical lung biopsy should be considered as the diagnostic procedures. Taken together, current invasive/intensive treatment has no definitive role in AE of IPF, while intensive treatment and methods of extensive diagnostics should be considered in the acute exacerbations of the other ILDs. However, in reality it is often very difficult to exclude infections of the IPF patients experiencing an acute deterioration, and therefore careful consideration is needed especially with those patients who may benefit from a lung transplantation operation.

## 7. Lung Biopsy in Mechanically Ventilated Patients

The lung biopsy procedure of mechanically ventilated patients has been previously assumed to carry a high risk for patients and thus it cannot be recommended [63–65]. However, recent studies have indicated, that lung biopsy operation is relatively safe, and it has been shown to be a rather secure procedure, even in mechanically ventilated patients [66–68]. Lim and co-authors studied 36 mechanically ventilated patients with undiagnosed diffuse pulmonary infiltrates who had undergone an open lung biopsy procedure for diagnostic purposes. The most common findings of the lung biopsies were IIPs, of which AIP was the most common type, occurring in 8 patients. Three of these patients had AE of IPF. In addition to the interstitial pneumonias, single cases of infections and drug reactions were also observed. The most common complication was a prolonged air leakage occurring in fifteen patients out of 36 (42%); none of the complications were fatal. The lung biopsy findings changed the treatments in 64% of the patients. If the open lung biopsy has been conducted within one week after the initiation of mechanical ventilation, there was improved prognosis of survival of the patients [69]. To conclude, lung biopsy can be recommended even in ventilated patients, but only after careful consideration and if accurate diagnosis is likely to contribute to the treatment.

## 8. Association of DAD with ARDS and Other Acute Reactions of the Lung

In 1967, Ashbaugh and co-authors described 12 patients with acute respiratory distress [70]. A new definition of lung injury was recommended in 1994 in which these patients were separated into two groups, namely, those patients with less severe hypoxemia being classified as having acute lung injury (ALI), and those with more severe hypoxemia as suffering from ARDS [71, 72]. The most common morphologic pattern of ARDS is DAD, the features of which correlate better with the phases of alveolar damage than with its specific cause. Another histological pattern, which can be clinically and radiologically expressed as ARDS, is AFOP. This pattern does not meet the strict histological criteria for either organizing pneumonia (OP) or DAD and the prognosis of AFOP is better than DAD. DAD has been divided into exudative, proliferative, and fibrotic, or organizing phases [73, 74]. The pathologic alterations seen in ARDS, however, have been shown to be heterogeneous, since in a recent study only half of the patients with ARDS displayed typical DAD in postmortem examination [75]. On the other hand, only less than half of patients with postmortem autopsy findings of DAD that were treated in intensive care unit had been diagnosed clinically as ARDS [76]. Significant differences between ARDS and AIP are supported by the large differences of DAD in the lungs of ARDS patients, and the known etiological trigger in ARDS but not in AIP, which are factors that may account for the variable patient recovery. 

A retrospective study on 58 patients with DAD lung histopathology revealed that 60% of them were immunocompromised and 90% of them fulfilled the ARDS criteria at the time of the lung biopsy [77]. The causes of DAD in that particular study were infections (22%), AIP (21%), non-infectious complication of an organ or stem cell transplantation (17%), CVD (16%), AE of IPF (12%), drug reactions (10%) and radiation therapy (2%). The overall mortality for the lung diseases was 53%, with the exception of AE of IPF, in which the mortality was higher, being 86%. The histology of DAD is not able to reveal the cause of alveolar damage, although a recent study depicted two forms of DAD in patients with ARDS showing that interstitial myofibroblast proliferation was more common in patients with chemotherapy or drug toxicity than with severe infections [78]. From the clinical point of view, it is important to understand that the histological pattern of DAD is a non-specific lung reaction, and it does not provide any information to the clinician about the reason for the disease, that is, sepsis, trauma, aspiration, infection, drugs or it can be associated with an idiopathic disease such as AIP.

## 9. Viral Infections, DAD and IPF

DAD is a common lung reaction in patients suffering influenza infection. A landmark paper by Goodpasture and co-authors described the autopsy findings of two patients who had suffered from influenza in 1918. In both of the cases, the lungs showed features of DAD including “hyaline materials upon the walls of dilated alveolar ducts and in alveoli” which is the typical histological pattern of DAD. In that particular paper, it was stated that “Both presented lesions in the lung which are to be regarded as peculiar to the pulmonary inflammation of influenza, yet no micro-organisms were demonstrated by the usual methods in the lungs of either” [79]. In avian influenza A H5N1 virus infection and severe acute respiratory syndrome (SARS), DAD is a common autopsy finding in the lungs [80–82]. A very recent study described the autopsy findings of 21 patients with H1N1-influenza A infection. The age of the patients varied from 1 to 68 years, with 72% being between 30–59 years. Twenty patients had DAD in their lungs, and in addition to DAD, the lungs of 6 patients exhibited also necrotizing bronchiolitis and a further five patients displayed extensive hemorrhage [83]. Overall, the most extensive manifestations of H1N1-influenza will be observed in the lungs and there is a risk for the development of DAD.

Several studies over decade ago have also suggested the role of viral infections in the pathogenesis of IPF; the viruses include hepatitis C [84], Ebstein Barr [85], cytomegalo, and other herpes viral infections [86]. Interestingly recent study by Vannella and co-workers hypothesized that gammaherpes viruses can serve as initiating cofactors in the pathogenesis of pulmonary fibrosis and even its AEs [87]. In line with these new findings our recent study identified that ELMOD2, a candidate gene for IPF, regulates antiviral responses [88]. It is highly likely that the role of viral infections in the etiology and pathogenesis of IPF and other interstitial lung diseases and their AEs may be more important than previously assumed.

## 10. Conclusions

Several idiopathic and nonidiopathic interstitial lung diseases display similar morphological features, namely DAD, either in the stable phase or especially during an exacerbation of the disease (Table 4). In IPF, NSIP, CVD-ILD, and allergic alveolitis, acute exacerbations may occur, in which the typical histological features of the disease are superimposed on DAD pathology. In addition to the IIPs including AIP, a clinical ARDS is also a DAD disease in its pathological features. Moreover, the most serious manifestations of influenza viral infections are DAD reactions within the lungs, and recent studies have suggested that viruses may play role in IPF and its exacerbations. Thus, it seems that in many ways, the DAD reaction in the lungs may be traced to many causes, and therefore it might be assumed to represent a serious nonspecific reaction in response to variety of triggers. Many factors in the pathogenesis of DAD remain still unresolved. If one could understand the mechanisms underlying the DAD reaction, it might be possible to use this novel information to develop improved treatment for many lung diseases, which today are untreatable. One of the major findings in these reactions is the injury to alveolar epithelium, and therefore therapeutic strategies aimed at protecting the epithelium may represent novel strategies that should be carefully evaluated in the future.

## 11. Future Perspectives

It is evident that interstitial lung diseases represent a very heterogenous group of lung diseases, including lung diseases with known reasons such as allergic alveolitis, connective tissue diseases associated with lung diseases and infectious lung disorders and in all of these the acute reaction within the lung may be similar, namely, DAD. This reaction can be postulated as representing one of the fundamental injurious responses of the lung. However, the pathogenetic mechanisms underlying DAD are still mostly unknown, especially in humans. Most experimental studies have been conducted with animals exposed to toxins, lipopolysaccharide, and so forth, and these reactions cannot be directly extrapolated to human lung since lung reactions occurring in mice and rats do not resemble exactly those encountered in human lung. 

Another limitation for studying DAD in human lung is the rarity of those diseases. It is very difficult, virtually impossible, to gather large numbers of patients/samples of DAD-associated lung diseases during their acute exacerbations without international collaboration involving several specialities. It can be argued that the study of DAD-associated lung diseases is even more difficult than that of IIPs or other types of relatively rare lung diseases, because DAD is confined to unpredictable periods of various diseases. A systematic collection of human lung tissue and cell material would significantly improve the clinical-radiological-histopathological interpretation especially if combined with experimental in vitro studies in cells established from human lung. 

Methods to study disease mechanisms have dramatically developed during the last decade, while some older statements and conclusions may require new interpretation and re-evaluation. For example recent evidence has suggested that viruses may play an important role not only in the aetiology and pathogenesis of ILDs but also in their exacerbations, and that viruses can directly cause DAD reaction in the lung. 

There is need for international efforts and collaborations to study DAD-associated lung diseases and to discover targeted therapies, since the diseases are uncommon, prognosis of these patients has been dismal for years and is still very poor [29], and the patients are being evaluated and treated at many specialities.

## Figures and Tables

**Table 1:** Classifications of interstitial pneumonias.

Liebow 1969	Katzenstein & Myers 1998	ATS & ERS Statement 2002	ATS & ERS Statement 2002
		Histological pattern	Clinico-radiographic-pathological diagnosis
UIP	UIP	UIP	IPF
DIP	DIP	DIP	DIP
	RB-ILD	RB-ILD	RB-ILD
LIP		LIP	LIP
GIP			
BIP		OP	COP
	AIP	DAD	AIP
	NSIP	NSIP	NSIP

UIP: usual interstitial pneumonia

IPF: idiopathic pulmonary fibrosis

DIP: desquamative interstitial pneumonia

RB-ILD: respiratory bronchiolitis interstitial pneumonia

LIP: lymphocytic interstitial pneumonia

GIP: giant cell interstitial pneumonia

BIP: bronchiolitis obliterans interstitial pneumonia

OP: organizing pneumonia

COP: cryptogenic organizing pneumonia

AIP: acute interstitial pneumonia

DAD: diffuse alveolar damage

NSIP: nonspecific interstitial pneumonia.

**Table 2:** Idiopathic interstitial pneumonias that may behave acutely.

	IPF	AIP	NSIP
Age/sex	50–70 years, more common in males	All ages, as common in females than in males	40–60 years, also in children. More common in females

Survival	Most of the patients had died after 2–4 years after of diagnosis	In-hospital mortality 12%–50%, 70% mortality in 6 months	5 year survival 74%–82 %

Course of disease	Usually slowly progressive over within years, sometimes rapidly, acute exacerbations may occur	Acute lower respiratory tract illness within 60 days	Stable or slowly progressing, acute exacerbations may occur

Treatment	No currently known efficient treatment.	Corticosteroids Immunosuppressants	Corticosteroids Immunosuppressants

Radiology	Honeycombing, reticulation, bronchiectasis and bronchiolectasis. Ground glass opacities and consolidations in AE.	Diffuse ground glass opacities and infiltrates	Ground glass opacities. No or scant honeycombing

Histopathology	Heterogenous appearance with areas of normal parenchyma, fibrosis and honeycomb cysts, which is termed as UIP. AE of UIP show also DAD in addition to UIP.	DAD with hyaline membranes, öedema and interstitial acute inflammation in acute phase. In organizing phase alveolar septal thickening with organizing fibrosis, type II pneumocyte hyperplasia, and patchy or diffuse airspace organization.	In cellular type of NSIP, chronic interstitial inflammatory cells involves alveolar walls, fibrotic form may include more advanced fibrosis. The histology is usually uniform. In AE of NSIP also DAD is present.

**Table 3:** Proposed criteria of AE of IPF by Collard and the IPF Clinical Research Network Invertigators [34].

Previous or concurrent diagnosis of IPF	
Unexplained worsening or development of dyspnea within 30 days	
HRCT with new bilateral ground-glass abnormality and/or consolidation superimposed on a background reticular or honeycomb pattern consistent with UIP pattern	
No evidence of pulmonary infection by endotracheal aspirate or bronchoalveolar lavage	
Exclusion of alternative causes, including the following: left heart failure, pulmonary embolism, identifiable cause of acute lung injury	

**Table 4:** Associations of DAD with interstitial lung diseases and severe lung reactions (reference number in parentheses).

AIP	[28]
AE of IPF	[33, 35, 44]
AE of NSIP	[39]
AE of CVD associated UIP and NSIP	[39]
AE of allergic alveolitis	[52]
ARDS	[75, 76]
- Infections	
- Organ or stem cell transplantation	
- Drug reaction	
- Radiation therapy	
Viral lung infections	[79]
- SARS	[80, 81]
- H1N1-influenza A	[83]

AE: acute exacerbation

UIP: usual interstitial pneumonia

IPF: idiopathic pulmonary fibrosis

NSIP: nonspecific interstitial pneumonia

CVD: collagen vascular disease.
